# An Atypical Epigenetic Mechanism Affects Uniparental Expression of Pol IV-Dependent siRNAs

**DOI:** 10.1371/journal.pone.0025756

**Published:** 2011-10-07

**Authors:** Rebecca A. Mosher, Ek Han Tan, Juhyun Shin, Robert L. Fischer, Craig S. Pikaard, David C. Baulcombe

**Affiliations:** 1 Department of Plant Sciences, University of Cambridge, Cambridge, United Kingdom; 2 School of Plant Sciences, University of Arizona, Tucson, Arizona, United States of America; 3 Biology Department, Indiana University, Bloomington, Indiana, United States of America; 4 Department of Plant and Microbial Biology, University of California, Berkeley, California, United States of America; Temasek Life Sciences Laboratory, Singapore

## Abstract

**Background:**

Small RNAs generated by RNA polymerase IV (Pol IV) are the most abundant class of small RNAs in flowering plants. In *Arabidopsis thaliana* Pol IV-dependent short interfering (p4-si)RNAs are imprinted and accumulate specifically from maternal chromosomes in the developing seeds. Imprinted expression of protein-coding genes is controlled by differential DNA or histone methylation placed in gametes. To identify epigenetic factors required for maternal-specific expression of p4-siRNAs we analyzed the effect of a series of candidate mutations, including those required for genomic imprinting of protein-coding genes, on uniparental expression of a representative p4-siRNA locus.

**Results:**

Paternal alleles of imprinted genes are marked by DNA or histone methylation placed by DNA METHYLTRANSFERASE 1 or the Polycomb Repressive Complex 2. Here we demonstrate that repression of paternal p4-siRNA expression at locus 08002 is not controlled by either of these mechanisms. Similarly, loss of several chromatin modification enzymes, including a histone acetyltransferase, a histone methyltransferase, and two nucleosome remodeling proteins, does not affect maternal expression of locus 08002. Maternal alleles of imprinted genes are hypomethylated by DEMETER DNA glycosylase, yet expression of p4-siRNAs occurs irrespective of demethylation by DEMETER or related glycosylases.

**Conclusions:**

Differential DNA methylation and other chromatin modifications associated with epigenetic silencing are not required for maternal-specific expression of p4-siRNAs at locus 08002. These data indicate that there is an as yet unknown epigenetic mechanism causing maternal-specific p4-siRNA expression that is distinct from the well-characterized mechanisms associated with DNA methylation or the Polycomb Repressive Complex 2.

## Introduction

Mendelian laws of inheritance state that a genetic element behaves identically when transmitted through maternal or paternal gametes. Genetic elements that break this law by exhibiting preferential or exclusive expression when inherited from one parent are genomically imprinted. Genomic imprinting is well described only in placental mammals and flowering plants, although a number of parent-of-origin-dependent effects are observed in other organisms [1], [2], [3], [4], [5].

Flowering plants are characterized by double fertilization, whereby two identical haploid sperm cells in the pollen grain fertilize two cells in the female gametophyte. Fertilization of the haploid egg cell generates the diploid embryo while fertilization of the diploid central cell generates the triploid endosperm. The endosperm is functionally analogous to mammalian placenta, acting as a conduit between maternal somatic tissues and the growing embryo but not contributing genetically to the next generation. Endosperm makes up the bulk of grains such as rice, wheat, and maize, making it a critical tissue for human nutrition. With a single exception in maize [6], all characterized imprinted genes in plants display uniparental expression specifically in the endosperm and some imprinted genes affect the growth and development of this tissue [7], [8].

In plants, imprinted genes are associated with hypomethylated maternal DNA regardless of which allele is expressed. In *Arabidopsis thaliana* differential DNA methylation is established by the opposing actions of DNA METHYLTRANSFERASE 1 (MET1) in the paternal gametophyte and the DNA glycosylase DEMETER (DME) in the central cell of the female gametophyte [9], [10]. Loss of paternal DNA methylation through mutation of *MET1* activates the normally silent paternal allele of *FLOWERING WAGENINGEN* (*FWA*), *FERTILIZATION INDEPENDENT ENDOPSERM 2* (*FIS2*) and *MATERNALLY EXPRESSED PAB C-TERMINAL* (*MPC*), and reduces expression of the paternal-specific imprinted gene *PHERES* (*PHE*) [11], [12], [13], [14]. Similarly, loss of DME activity inhibits the maternal expression of at least *FIS2* and *MPC* [13], [15], and ectopic expression of *DME* outside of the central cell is sufficient to induce expression of another maternal-specific gene, *MEDEA* (*MEA*) [16]. These observations demonstrate the importance of DNA methylation patterns in the expression of imprinted genes.

Over 125 genes in *Arabidopsis* are imprinted [17]. In contrast, thousands of intergenic regions producing RNA Polymerase IV-dependent small interfering (p4-si) RNAs are maternally expressed in the developing seed [18]. Many p4-siRNAs are produced from transposable elements, but others coincide with imprinted genes such as *FWA*, *MPC*, and *MEA* [19], [20] indicating that there may be a connection between parent-of-origin specific expression of protein-coding genes and non-coding RNAs. Recent genome-wide analyses of DNA methylation in the endosperm further support this connection between p4-siRNA expression and imprinting of genes because maternal chromosomes undergo extensive DME-mediated DNA demethylation at regions of p4-siRNA production [21], [22]. A plausible scenario was that p4-siRNAs and imprinted genes might be coordinately regulated by DME and MET1.

To examine the mechanism of p4-siRNA imprinting we investigated the genetic requirements for maternal expression and paternal silencing of p4-siRNAs at a representative locus. Here we show that differential DNA methylation does not explain uniparental expression of p4-siRNAs at locus 08002 and that various histone modifications, including Histone H3 Lysine 27 methylation (H3K27me), do not establish maternal-specific expression at this site. Furthermore, demethylation of maternal chromosomes by DME is dispensable for p4-siRNA expression in the endosperm.

## Results

### Loss of DNA methylation does not alter uniparental p4-siRNA expression in endosperm

**Figure 1:**
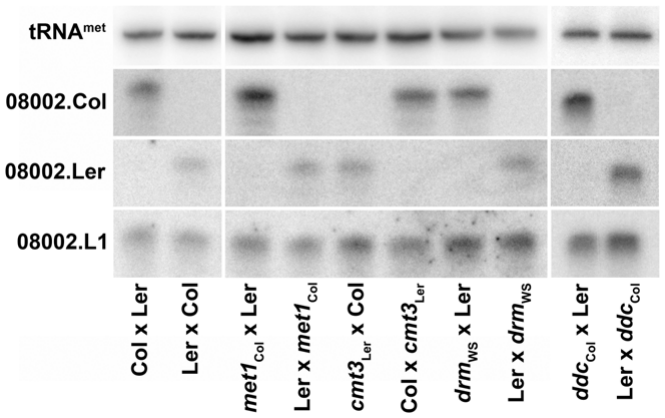
Loss of methylation does not induce biparental p4-siRNA production in endosperm. Small RNAs were isolated from inter-ecotype crosses between wild type and DNA methyltransferase mutants at 5 days after fertilization; maternal parent is listed first for all crosses. Parental origin of small RNA was determined with allele-specific small RNA probes (08002.Col and 08002.Ler). 08002.L1 hybridizes to small RNAs from both alleles and is a control for small RNA production at this locus; tRNA^met^ is a loading control. Small RNAs were detected specifically from maternal alleles in crosses between the wild-type ecotypes Columbia-0 (Col) and Landsberg *erecta* (Ler). Demethylation of the paternal genome through the mutations *dna methyltransferase 1* (*met1*), *chromomethyltranserase 3* (*cmt3*), and *domains rearranged methyltransferases 1* and 2 (*drm*) was not sufficient to trigger accumulation of paternal p4-siRNAs. Furthermore, loss of all non-CG methylation in the triple mutant *drm1 drm2 cmt3* (*ddc*) was insufficient to trigger paternal p4-siRNA accumulation.

Loss of DNA METHYLTRANSFERASE 1 (MET1) does not alter maternal-specific expression of p4-siRNAs at locus 08002 in *Arabidopsis* endosperm [18]. MET1 is the primary methyltransferase in *Arabidopsis* and is responsible for maintenance of CG dinucleotide methylation [23]. Methylation at CHG sites (where H is A, T, or C) is performed by CHROMOMETHYLTRANSFERASE 3 (CMT3) and asymmetric methylation (at CHH sites) is placed by DOMAINS REARRANGED METHYLTRANFERASES (DRM1 and DRM2) [23]. To determine whether non-CG DNA methylation represses paternal p4-siRNA alleles or induces expression of maternal alleles, we crossed *cmt3* or *drm* mutants and wild-type plants of a differing ecotype. Five days after fertilization, when p4-siRNA accumulation is highest, we extracted RNA from crossed fruits and performed allele-specific northern blots to determine the parental origin of p4-siRNAs at locus 08002 (figure 1). Demethylation of the pollen donor was insufficient to induce paternal locus 08002 p4-siRNA accumulation, indicating that CHG and CHH methylation do not repress paternal expression of p4-siRNAs. In reciprocal crosses, no change in locus 08002 p4-siRNA accumulation was detected, indicating that non-CG methylation is not required for maternal p4-siRNA expression.

To determine whether CHG and CHH methylation might act redundantly to repress expression, as occurs at *SUPPRESSOR OF drm1 drm2 cmt3* [24], we also used the *drm1 drm2 cmt3* triple mutant (*ddc*) as maternal or paternal parent in inter-ecotype crosses. Maternal-specific expression of locus 08002 p4-siRNAs was maintained even when the pollen donor lacked both CHG and CHH methylation (figure 1). These results indicate that differential DNA methylation is not responsible for maternal-specific accumulation of p4-siRNAs at locus 08002 although we have not ruled out that MET1 acts redundantly with either CMT3 or DRM proteins.

### Various chromatin modifications do not affect p4-siRNA expression

**Figure 2:**
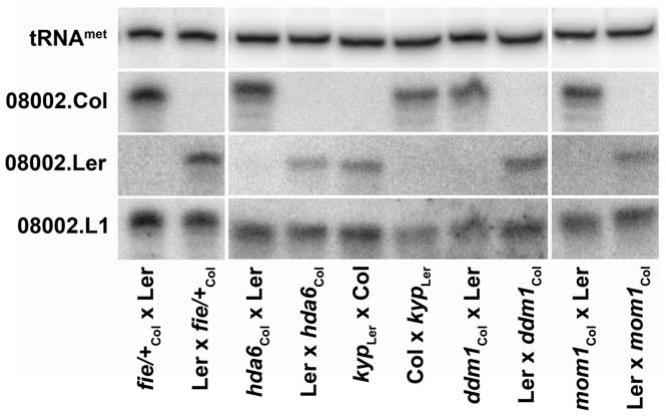
Assorted chromatin modifications are not required for imprinted p4-siRNA production in endosperm. Small RNAs were isolated from inter-ecotype crosses between wild type and a histone modification mutant and parental origin of small RNA was determined as described in figure 1. Accumulation of p4-siRNAs from paternal chromosomes was not induced when the Polycomb Repressive Complex 2 mutant *fertilization independent endosperm* (*fie*) was transmitted paternally. Likewise, mutations in *histone deacetylase 6* (*hda6*), the H3K9 methyltransferase *kryptonite* (*kyp*), and the nucleosome remodeling proteins *decrease in dna methylation 1* (*ddm1*) and *morpheus' molecule 1* (*mom1*) did not affect uniparental expression of p4-siRNAs.

The *Arabidopsis* gene *MEDEA* (*MEA*) is exceptional because, unlike other imprinted genes, paternal expression is not repressed by MET1-mediated DNA methylation, but rather by histone H3 lysine 27 methylation (H3K27me) placed by the Polycomb Repressive Complex 2 (PRC2). Loss of PRC2 in the pollen or female gametophyte triggers biparental expression of *MEA* in developing endosperm [12]. To investigate the role of PRC2 in uniparental expression at locus 08002, we performed crosses as above with the PRC2 mutation *fertilization independent endosperm* (*fie*). FIE is the only *Extra Sex Combs* homolog in *Arabidopsis* and this mutation lacks all potential PRC2 complexes [7]. When *fie* is transmitted through pollen (from a heterozygous pollen donor) paternal *MEA* accumulates in the developing seeds [12]. However, biparental expression of locus 08002 p4-siRNAs was not detected when this mutation was present in the paternal lineage (figure 2).

To determine if other chromatin modifications might repress paternal expression of p4-siRNAs at locus 08002, we tested several candidate genes as above. *HISTONE DEACETYLASE 6* (*HDA6*) is associated with silencing of transposable elements [25] and rDNA repeats [26], [27]. *KRYPTONITE* (*KYP*) encodes a histone methyltransferase that catalyzes dimethylation at lysine 9 of histone H3 (H3K9me), the canonical mark of silent chromatin [28]. *DECREASE IN DNA METHYLATION 1* (*DDM1*) and *MORPHEUS' MOLECULE 1* (*MOM1*) have similarity to SWI2/SNF2 ATPases and encode presumed nucleosome remodeling proteins. Mutations in *DDM1* eliminate DNA methylation and transcriptional silencing from transposable elements [25], [29], while loss of *MOM1* causes transcriptional reactivation of transgenes and repeated sequences without changes in DNA methylation [30], [31]. These factors were not necessary for either paternal repression or maternal expression of locus 08002 p4-siRNAs in the endosperm (figure 2).

### DEMETER-family glycosylases are neither sufficient nor necessary to induce p4-siRNA expression

Loci generating p4-siRNAs undergo extensive DME-mediated DNA demethylation in the central cell, leading to differential methylation in endosperm [21], [22]. Demethylation by DME is required for expression of *MEA*, *FWA*, and *FIS2* [10], [11], [15], and partially required for expression of *MPC* [13]. *DME* expression is also sufficient for expression of at least *MEA*, as ectopic expression of *DME* triggers *MEA* accumulation in vegetative tissue and from paternal alleles [16]. DME is part of a small family of glycosylases in *Arabidopsis* including REPRESSOR OF SILENCING (ROS1), a protein implicated in maintaining the expression of transgenes [32], and two related proteins, DEMETER-LIKE 2 (DML2) AND DEMETER-LIKE 3 (DML3) [33].

**Figure 3:**
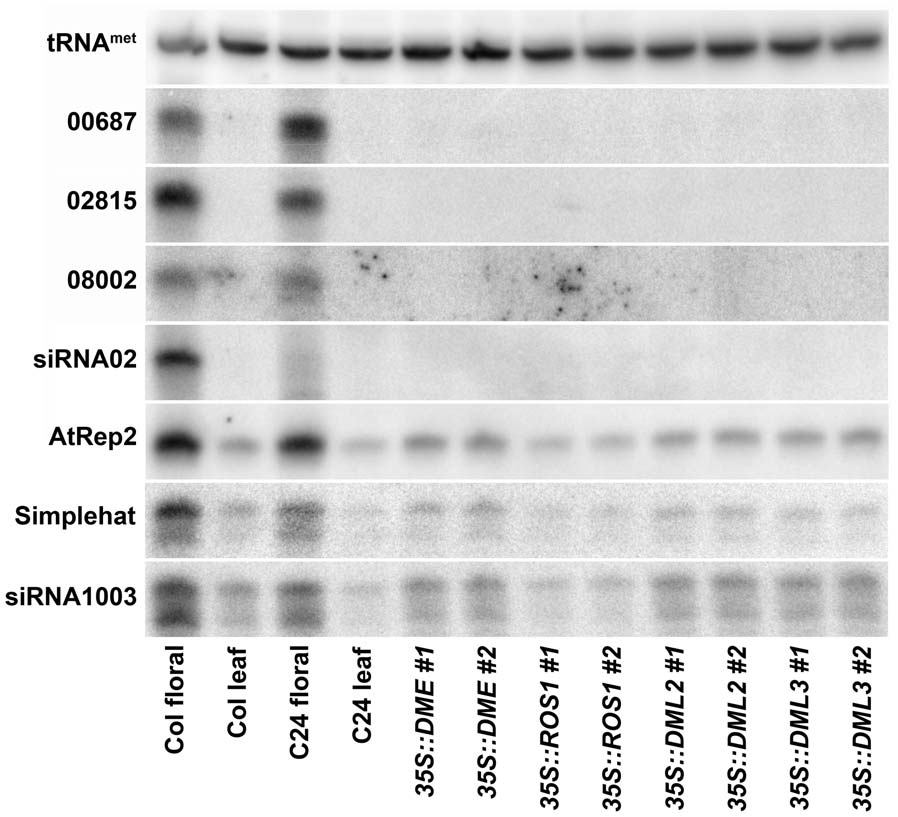
*DEMETER* family glycosylases are insufficient to induce vegetative expression of p4-siRNAs. Ectopic expression of the *DEMETER* glycosylase behind the strong, nearly constitutive *35S* promoter (*35S::DME*) does not cause ectopic accumulation of type I p4-siRNAs (00687, 02815, 08002, and siRNA 02) in leaves, nor does it alter expression of type II p4-siRNAs (AtRep2, Simplehat, and siRNA1003) in leaves. Similarly, overexpression of the related glycosylases *REPRESSOR OF SILENCING* (*35S::ROS1*), *DEMETER-LIKE 2* (*35S::DML2*), or *DEMETER-LIKE 3* (*35S::DML3*) has no affect on p4-siRNA expression. Two independent transgenic lines were assayed for each overexpression construct. *35S::ROS1* lines are in the C24 background [34]; all other lines are in the Col background [16].

To determine whether demethylation by DME or its relatives is involved in maternal expression of p4-siRNAs, we first assayed p4-siRNA expression in transgenic lines overexpressing each glycosylase behind the nearly constitutive *35S* promoter (figure 3, figure S1) [16], [34]. Independent transgenic lines did not display ectopic expression of type I p4-siRNAs, which are normally restricted to endosperm, nor did they enhance expression of type II p4-siRNAs, which accumulate vegetatively [18]. These observations indicate that demethylation by DME or its relatives are not sufficient to trigger p4-siRNA expression.

**Figure 4:**
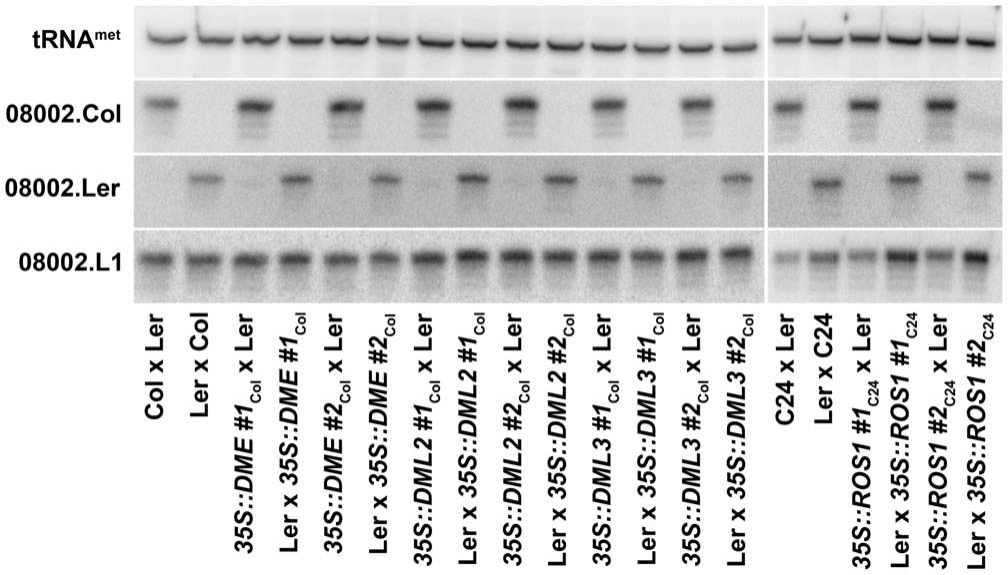
*DEMETER* family glycosylases do not trigger paternal expression of p4-siRNAs. Small RNAs were isolated from inter-ecotype crosses between wild type and transgenic lines and parental origin of small RNA was determined as described in figure 1. Expression of the *DEMETER* glycosylase in the male gametophyte from the strong, nearly constitutive *35S* promoter (*35S::DME*) does not trigger paternal expression of p4-siRNAs in endosperm. Similarly, overexpression of the related glycosylases *REPRESSOR OF SILENCING* (*35S::ROS1*), *DEMETER-LIKE 2* (*35S::DML2*), or *DEMETER-LIKE 3* (*35S::DML3*) does not affect imprinted p4-siRNA expression in endosperm. Two independent transgenic lines were assayed for each overexpression construct. *35S::ROS1* lines are in the C24 background [34]; all other lines are in the Col background [16].

To determine whether demethylation acts in conjunction with endosperm-specific factors to trigger expression of p4-siRNAs, we next crossed DME family overexpression lines to wild-type plants of a different ecotype and determined parental origin of locus 08002 p4-siRNAs at 5 days after fertilization. If demethylation is required for expression, crosses generated with the transgenic lines as pollen donors should result in biallelic expression of p4-siRNAs. Instead, strict maternal-specific expression was detected for all crosses (figure 4), indicating that ectopic demethylation of the paternal genome by overexpression of DME family glycosylases is insufficient to induce paternal expression at locus 08002.

**Figure 5:**
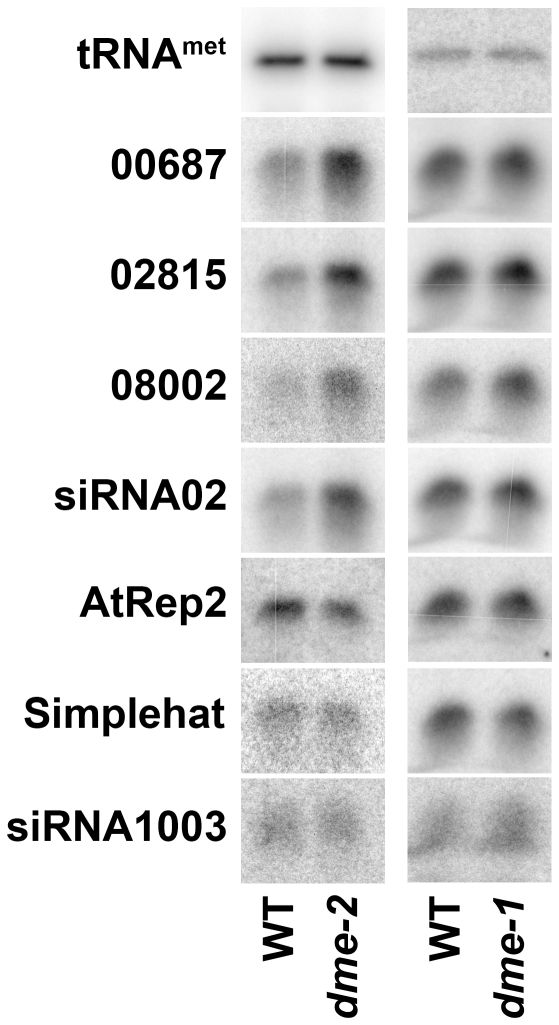
p4-siRNA expression in endosperm does not require DEMETER demethylation. Left side: Developing (WT) or arrested (dme-) seeds were dissected from self-fertilized *dme-2* heterozygous fruits 10–12 days after fertilization and small RNAs were extracted. DME-deficient seeds express p4-siRNAs at levels higher than wild type, perhaps due to arrest at an earlier developmental stage or due to endosperm overgrowth. Right side: RNA was extracted from wild type and *dme-1* homozygous fruits at 5 days after anthesis and small RNAs were extracted. Mutant seeds accumulate p4-siRNAs slightly higher than wild type seeds, perhaps due to endosperm overgrowth in mutant seeds.

To further assess the role of DME in accumulation of p4-siRNAs, we assayed p4-siRNA expression in *dme* mutant endosperm, which is not demethylated at p4-siRNA loci [21], [22]. In *dme-2* heterozygotes, seeds inheriting a maternal *dme* allele abort early in development while seeds inheriting a maternal *DME* allele develop normally. To determine whether DME is necessary for accumulation of p4-siRNAs from maternal chromosomes, we dissected aborted and developed seeds from heterozygous *dme-2* self-fertilized siliques during mid-embryo development (10–12 days post-fertilization). Unexpectedly, p4-siRNA accumulation in *dme* seeds was higher than in wild-type siblings (figure 5). To determine whether this was due to lack of demethylation by DME or due to the developmental arrest of mutant seeds during an earlier period of high p4-siRNA accumulation, we analyzed wild-type and *dme* seeds from the same developmental stage. When transmitted maternally the weaker *dme-1* allele does not always trigger seed abortion, making homozygous mutant lines possible. Developing siliques from *dme-1* and wild type were collected at 5 days post anthesis and p4-siRNA accumulation was assayed (figure 5). *dme-1* siliques display wild-type expression of p4-siRNAs, indicating that demethylation by DME is not necessary for p4-siRNA production from maternal chromosomes in the endosperm.

## Discussion

Differential methylation of maternal and paternal DNA is extensive in the endosperm of *Arabidopsis*, primarily due to DEMETER-mediated demethylation of transposable elements in the central cell [21], [22]. Many transposable elements produce p4-siRNAs, leading to the hypothesis that demethylation of these elements in the endosperm causes maternal-specific production of p4-siRNAs [22]. However, loss of DNA methylation in developing seeds is insufficient for paternal p4-siRNA expression from the representative p4-siRNA locus 08002 (figure 1), and loss of maternal DNA demethylation does not eliminate p4-siRNA expression (figure 5). We also demonstrate that several known histone modifications, including H3K27 and H3K9 methylation, are dispensable for paternal repression of locus 08002 (figure 2). These data indicate an additional, as yet uncharacterized chromatin signal affects maternal expression and paternal repression of 08002 and possibly other p4-siRNA loci in developing seeds.

Evidence indicates that this activating mark is established before fertilization because p4-siRNA expression in the maternal flowers is required for p4-siRNA expression in the developing seed [18]. Lack of p4-siRNA expression in mature pollen would restrict this activating mark to maternal chromosomes [35]. This unidentified mark could also be used by protein-coding genes because many maternal-specific transcripts in the *Arabidopsis* seed transcriptome identified are unaffected by loss of DNA or H3K27 methylation [17].

We had previously concluded, based on dissection and genetic analysis, that the activating mark would be carried on the maternal alleles of the fertilized endosperm. This remains a plausible explanation. However there is also the possibility that the maternal p4-siRNAs are expressed in the maternal seed coat and transported into the endosperm. A formal additional possibility is that these RNAs are present in the seed coat associated with our dissected endosperm. However these explanations based on sporophytic expression are only consistent with our genetic analysis if *NRPD1* activity is affected by gene dosage. Therefore we favor the hypothesis based on specific expression from the maternal alleles in endosperm.

Although there is significant overlap between regions of DME demethylation and p4-siRNA expression [21], [22], we have shown that DME demethylation is not required for p4-siRNA expression at a variety of p4-siRNA loci. Furthermore, p4-siRNA expression in the female gametophyte is not required for DME activity because none of the mutations that lack p4-siRNAs exhibit the seed abortion phenotypes associated with loss of DME activity. These data lead to the conclusion that DME-mediated DNA demethylation and p4-siRNA expression occur independently at many genomic regions, especially transposable elements. *FWA* is imprinted in *Arabidopsis halleri*, most likely through the action of DME at a SINE element, and yet *A. halleri FWA* lacks the tandem repeats that are required for p4-siRNA expression in *A. thaliana* [19], [36]. *A. halleri FWA* might therefore be an example of a genomic region that has recruited DME but not Pol IV. It is possible that DME and Pol IV have independent roles in establishing parent-of-origin chromatin signatures across the *Arabidopsis* genome.

Parent-of-origin chromatin signals might be more prevalent than previously thought. Although imprinted expression of endogenous protein-coding genes has only been described in placental mammals and flowering plants, parent-of-origin phenomena exist throughout the animal kingdom. Some transgenes in the nematode *Caenohabditis elegans* and the zebrafish *Danio rerio* are imprinted [1], [5], and *Drosophila melanogaster* transgenes inserted near regions of heterochromatin or within the Y chromosome are also imprinted [2], [37]. Parent-of-origin effects are not limited to uniparental gene expression. The first published case of parental “imprints” is Sciarid flies, where paternal chromosomes are eliminated from specific cell lineages [3], [38]. In coccid insects the entire paternal genome is either heterochromatinized or eliminated from somatic tissues [4], while in *C. elegans* the X chromosome adopts specific histone modifications depending on the parent of origin [39]. It seems likely that parent-of-origin chromatin signatures are widespread throughout sexual eukaryotes, and it will be interesting to discover what role small RNA-directed chromatin modification might play in establishing or responding to these signals.

## Materials and Methods

### Plant growth conditions and genotypes

All plants were grown under standard conditions including 16 hours of light each day. Mutant alleles were as follows. Columbia ecotype: *met1-1* [40], *drm 1-2* (SALK_031705) [41], *drm2-2* (SALK_150863) [41], *cmt3-11* (SALK_148381) [41], *hda6-9* (E. Havecker, C. Melnyk, and D. Baulcombe, unpublished allele), *ddm1-2* [42], *mom1-2* (SALK_141293) [30], and *fie* (GABI 362D08); Landsberg *erecta* ecotype: *cmt3-7* [43], and *kyp-2* [28]; Wassilewskijia ecotype: *drm1-1* [44] and *drm2-1* [44]. The *dme-1* and *dme-2* mutations were isolated in Columbia and backcrossed to Landsberg *erecta* [16]. The *drm1 drm2* double mutant contained *drm1-1* and *drm2-1*; the ddc triple mutant contained *drm1-2*, *drm2-2*, and *cmt3-11*. Mutations were confirmed using molecular markers or visible phenotypes. Wassilewskijia and C24 contain the Columbia-0 allele at locus 08002 (figure S2).

To eliminate possible self-fertilization, crosses were performed 24 hours after manual emasculation of immature flowers. For each cross, six to ten siliques were collected 5 days after fertilization. To determine the effect of the loss-of-function *dme-2* allele, *dme-2* heterozygotes were allowed to self-fertilize. The resulting seeds were dissected 10–12 days after fertilization and divided into DME+ and dme- based on development of the embryo. For analysis of the weaker *dme-1* allele, flowers were inspected daily and marked upon anthesis. Siliques were collected 5 days after anthesis.

### Generation of transgenic lines

Total RNA from wild-type Columbia-0 leaf tissue was used to reverse transcribe and amplify full-length cDNAs of DML2 and DML3 with the following primers: DML2: 5′-CACCATGGAAGTGGAAGGTGAAGTG-3′ and 5′-TCATTCCTCTGTCTTCTCTTTAGTTCTG-3′; DML3 5′-CACCATGTTGACAGATGGTTCACAACAC-3′ and 5′-CTATATATCATCATCACTCATAAACTTTGGCC-3′. PCR products were introduced into pENTR D-TOPO (Invitrogen) and the resulting entry vectors were recombined into pEARLEYGATE 202 [45]. 35S::DML2 and 35S::DML3 constructs were stably transformed into wild-type Columbia-0 using standard protocols. Generation of 35S::DME and 35S::ROS1 are described elsewhere [16], [34].

Overexpression of DME-family glycosylases was verified with quantitative reverse transcription-PCR using QuantiFast SYBR Green One-Step RT-PCR Kit (Qiagen) and the following primers: DME 5′-ATTAAGGATTTCCTAGAACG-3′ and 5′-ATCCTAACTGCTATCCTTCC-3′; MEA 5′-GCTAATCGTGAATGCGATCC-3′ and 5′-AGAGAGTCCCATGTAAATGC-3′; ROS1 5′-GGGATGAACCATAAACTTGC-3′ and 5′-CAACTGGAAAGGCAAGATGG-3′; DML2 5′-GCTTGCCGAAAGAATCAAGG-3′ and 5′-CCGACATTCGTGTCAACAGG-3′; DML3 5′-GAATGGCTTCGAAATGCTCC-3′ and 5′-GGTACTCGAATAGTTGATGC-3′; GAPDH 5′-CTCCCTTGGAAGGAGCTAGG-3′ and 5′-GATGCATTGCTGATGATAGG-3′ (figure S1).

### RNA extraction and northern hybridizations

RNA was extracted from leaves using TRI® Reagent (Sigma-Aldrich) according to the manufacturer's protocol. RNA from crossed siliques or dissected seeds was extracted as follows: 5–6 siliques were frozen in liquid nitrogen and ground to a fine powder. 500 µL of room temperature extraction buffer (100 mM glycine pH 9.5, 10 mM EDTA, 100 mM NaCl, 2% SDS) was added and once thawed, samples were further homogenized and placed on ice. Lysates were extracted once with cold Tris-saturated phenol (pH 8.0), twice with cold 25∶24∶1 Tris-saturated phenol∶chloroform∶isoamyl alcohol, and once with cold 24∶1 chloroform∶isoamyl alcohol before precipitation with sodium acetate and ethanol.

Small RNA was enriched from 30–50 µg total RNA with mirVana miRNA isolation columns (Ambion) according to the manufacturer's protocol. Small RNAs were resolved on a 7M urea/1X TBE/15% acrylamide gel (19∶1 acrylamide∶bisacrylamide) and transferred to Hybond N+ membrane (GE/Amersham). Membranes were UV-crosslinked before pre-hybridization in UltraHyb Oligo buffer (Ambion). Oligonucleotides were labeled with [γ-^32^P]-ATP and T4 polynucleotide kinase and purified over an illustra MicroSpin G-25 column (GE/Amersham). After overnight hybridization with labeled oligonucleotides in UltraHyb Oligo buffer membranes were washed twice in 2X SSC, 0.1% SDS. Hybridization and washing was at 35°C. Membranes were exposed to phosphor-storage screens for detection of siRNAs.

Probe sequences are as follows (underlined bases are LNA): tRNA^met^ 5′-TCGAACTCTCGACCTCAGGAT-3′; 08002.L1 5′-CCCATGGTCTCAAACACATCCTCG-3′; 08002.Ler 5′-TCAAGTGAATCTTTAGCGTATGCT-3′; 08002.Col 5′-AGTGAATCTAGAGATTTAGCGTAT-3′; 00687 5′-GTTCCTCGTTCTACCCTCATACCT-3′; 02815 5′-CCATGTCATTCCACCCATCAAAAG-3′; siRNA02 5′-GTTGACCAGTCCGCCAGCCGAT-3′; AtRep2 5′-GCGGGACGGGTTTGGCAGGACGTTACTTAAT-3′; Simplehat 5′-TGGGTTACCCATTTTGACACCCCTA-3′; siRNA1003 5′-ATGCCAAGTTTGGCCTCACGGTCT-3′. All experiments were replicated with independent biological samples.

## Supporting Information

Figure S1 **Characterization of** ***DEMETER*** **family overexpression lines.** Transgenic lines expressing the four members of the *DEMETER* family behind the nearly constitutive *35S* promoter were assayed for transcript accumulation in leaves by quantitiative reverse transcription-PCR. Overexpression of *REPRESSOR OF SILENCING* (*ROS1*) is in the C24 ecotype [34]; all other constructs are in Columbia (Col-0) [16]. All graphs are mean values for 3 biological replicates and were normalized to GAPDH expression. *35S::DME* and *35S::ROS1* lines are homozygous; *35S::DML2* and *35S::DML3* are pooled samples of homozygous and hemizygous T2 individuals. Overexpression of *DEMETER* (*DME*) is weak, but sufficient to induce expression of *MEDEA* (*MEA*) in leaves (pink bars).
(EPS)

Figure S2 **The 08002 polymorphism in various** ***Arabidopsis*** **ecotypes.** The p4-siRNA locus 08002 contains a six nucleotide indel between *Arabidopsis* ecotypes Columbia (Col) and Landsberg *erecta* (Ler). This polymorphism is the basis of the allele-specific probes 08002.Col and 08002.Ler (hybridizing to the region in bold type). To determine if these probes would also bind siRNAs from other ecotypes, the 08002 region from Wassilewskijia (WS) and C24 was sequenced. These ecotypes are (Col)-like for the indel, but they also differ from Col at a single nucleotide (in red). However, this SNP does not appear to affect hybridization of the Col probe to C24 and WS siRNAs.
(TIF)
