# Supplementary figures and images for: An Atypical Epigenetic Mechanism Affects Uniparental Expression of Pol IV-Dependent siRNAs
Source: PLoS One. 2011 Oct 7;6(10):e25756. doi: 10.1371/journal.pone.0025756 (PMC3189211; doi:10.1371/journal.pone.0025756)

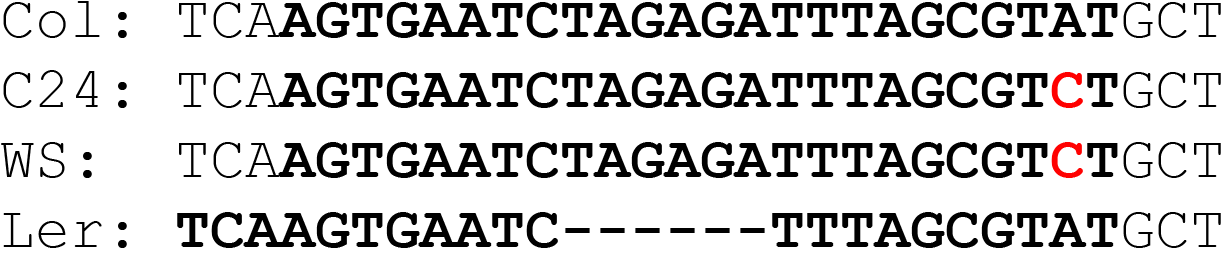

Supplement: Figure S2 — The 08002 polymorphism in various Arabidopsis ecotypes. The p4-siRNA locus 08002 contains a six nucleotide indel between Arabidopsis ecotypes Columbia (Col) and Landsberg erecta (Ler). This polymorphism is the basis of the allele-specific probes 08002.Col and 08002.Ler (hybridizing to the region in bold type). To determine if these probes would also bind siRNAs from other ecotypes, the 08002 region from Wassilewskijia (WS) and C24 was sequenced. These ecotypes are (Col)-like for the indel, but they also differ from Col at a single nucleotide (in red). However, this SNP does not appear to affect hybridization of the Col probe to C24 and WS siRNAs. (TIF) [file pone.0025756.s002.tif]
